# Integrative multi-omics analysis reveals lipid/metabolite dysregulation and temporal decoupling in disease progression

**DOI:** 10.1038/s41598-026-70293-z

**Published:** 2026-09-08

**Authors:** Uchenna Alex Anyaegbunam, Vincent ten Cate, Katrin Bauer, Thierry Schmidlin, Ute Distler, Stefan Tenzer, Elisa Araldi, Laura Bindila, Philipp Wild, Miguel A. Andrade-Navarro

**Affiliations:** 1 Computational Biology and Data Mining group (CBDM), Institute of Organismic and Molecular Evolution (iOME), Johannes Gutenberg University, Mainz, Germany; 2 Preventive Cardiology and Preventive Medicine, Department of Cardiology, University Medical Center of the Johannes Gutenberg University Mainz, Langenbeckstr. 1, 55131 Mainz, Germany; 3 Clinical Epidemiology and Systems Medicine, Center for Thrombosis and Hemostasis (CTH), University Medical Center, Mainz, Germany; 4 Partner Site Rhine Main, German Center for Cardiovascular Research (DZHK), University Medical Center of the Johannes Gutenberg University Mainz, Mainz, Germany; 5 Computational Systems Medicine, Center for Thrombosis and Hemostasis (CTH), Mainz, Germany; 6 Institute of Immunology, University Medical Centre of the Johannes- Gutenberg University Mainz, Mainz, Germany; 7 Research Centre for Immunotherapy (FZI), University Medical Center of the Johannes-Gutenberg University Mainz, Mainz, Germany; 8 Helmholtz Institute for Translational Oncology Mainz (HI-TRON Mainz) - A Helmholtz Institute of the DKFZ, Mainz, Germany; 9 DKFZ German Cancer Research Center, Heidelberg, Germany; 10 Institute of Physiological Chemistry, University Medical Center, Mainz, Germany

**Keywords:** Lipids, Metabolites, Proteins, Chronic inflammation, Toxic inflammation, Multi-omics integration, Biochemistry, Diseases

## Abstract

Our study presents and applies a metabolomics-driven multi-omics integration strategy to elucidate dynamic pathway interactions during disease progression. We analyzed longitudinal metabolomics datasets from a Duchenne muscular dystrophy (DMD) mouse model (6–30 weeks) and an acute Bothrops asper envenomation model (1–24 h) to contrast chronic versus acute inflammation. In the DMD model, we predicted phased cross-talk between sphingolipid metabolism and neurotrophin signaling: an early proteomic surge followed by lipid-mediated amplification and a late convergence at the protein level. Arginine and proline metabolism exhibited early metabolite accumulation preceding delayed inferred protein changes, consistent with impaired nitric oxide synthesis and argininemia-like effect. We also predicted late-stage activation of the AGE–RAGE pathway in DMD, likely triggered by ceramide buildup, and an autophagy-related lipid metabolic shift at mid-stage. In the envenomation model, tryptophan–kynurenine and nicotinamide pathways for NAD⁺ biosynthesis were rapidly perturbed at the metabolite level (1–3 h) but induced corresponding predicted enzymes only by 24 h. Thyroid hormone signaling showed an early coupling of substrate availability (tyrosine surge at 1 h) with predicted stress-response proteins and a second, delayed wave of inferred transcriptional regulators at 24 h. Acute envenomation also triggered immediate glycine/serine utilization possibly for antioxidant defense and glycerophospholipid breakdown (via phospholipase A₂), whereas chronic DMD showed sustained glycine/serine engagement and inferred, unresolved phospholipid perturbation without protein-level compensation, which may result from chronic oxidative stress. Overall, our integrative analysis revealed time-specific, multi-layer molecular perturbations distinguishing acute toxin injury from chronic muscle degeneration. Key metabolic control points (ceramide accumulation, arginine flux diversion, autophagy–lipid cross-talk, NAD⁺ salvage timing) were identified, highlighting potential targets for stage-specific therapeutic or nutritional interventions.

**Supplementary Information:**

The online version contains supplementary material available at https://doi.org/10.1038/s41598-026-70293-z.

## Introduction

Chronic diseases are frequently characterized by a state of persistent low-grade inflammation that disrupts normal metabolic homeostasis^1–2^. This phenomenon, often termed “metaflammation” links obesity, type 2 diabetes, cardiovascular disease and other chronic conditions via common inflammatory pathways and cytokine-driven insulin resistance^3–8^. For example, pro-inflammatory mediators such as tumor necrosis factor-α and interleukin-6 are overproduced in obese adipose tissue and can interfere with insulin signaling, promoting systemic metabolic dysfunction^9–12^. Reciprocally, metabolic disorders exacerbate inflammation – elevated glucose and lipid levels can activate innate immune pathways, creating a vicious cycle of chronic inflammation and metabolic disturbance^12–17^. Recent longitudinal studies have even demonstrated that inflammation can precede and trigger metabolic syndrome, underscoring the primacy of inflammation as an initiator in disease pathogenesis^18–20^. Together, these findings establish chronic inflammation as both a hallmark and driver of metabolic dysfunction in diverse chronic diseases. A major consequence of this pathologic cross-talk is dysregulated metabolite and lipid signaling that perpetuates inflammation. Inflammatory cytokines not only alter glucose and protein metabolism, but also profoundly impact lipid pathways, favoring accumulation of bioactive lipids that fuel inflammation^15,21–23^. Many lipid species function as potent mediators in the immune system: for instance, saturated fatty acids engage Toll-like receptors on macrophages, triggering NF-κB and inflammasome activation, while oxidized phospholipids and cholesterol derivatives can activate endothelial cells and leukocytes^24–25^. Conversely, the resolution of inflammation is also orchestrated by lipids – polyunsaturated fatty acid metabolites (e.g. resolvins) and other pro-resolving lipid mediators actively dampen inflammation and restore homeostasis^26–28^. Thus, dysfunctional lipid metabolism creates an imbalance between pro- and anti-inflammatory signals. Endogenous bioactive lipids such as eicosanoids, sphingolipids, and endocannabinoids are secreted by virtually all immune and metabolic cells and constitute a crucial molecular interface between metabolism and immunity^26,29–30^. In chronic disease, this interface is skewed toward pro-inflammatory lipid profiles: studies show that patients with Duchenne muscular dystrophy (DMD), obesity, diabetes, atherosclerosis, and other conditions have dysfunctional levels of pro-inflammatory lipid mediators and impaired generation of resolving lipids^31–34^. Bioactive lipids are now recognized not merely as bystanders of inflammation, but as active drivers that connect metabolic stress to immune activation^35–37^.

One class of lipids that exemplifies this maladaptive signaling is the sphingolipids, particularly ceramides. Ceramides have emerged as key contributors to metabolic inflammation, linking overnutrition to insulin resistance and tissue inflammation^38–41^. Excessive ceramide accumulation in metabolic tissues – as occurs in obesity and lipotoxic states – prompts adipose tissue macrophage infiltration and inflammasome activation, driving insulin resistance and inflammatory cytokine production^40–43^. Notably, ceramides and their metabolites can modulate multiple immune signaling pathways. Ceramide-1-phosphate (C1P), for example, directly activates cytosolic phospholipase A₂ and stimulates an “eicosanoid storm,” amplifying both acute and chronic inflammatory responses^44–47^. Likewise, sphingosine-1-phosphate (S1P) engages specific G-protein-coupled receptors on lymphocytes and endothelial cells to regulate immune cell trafficking and vascular inflammation^48–49^. Intriguingly, ceramides and C1P can also exert feedback inhibition on certain inflammatory signals – for instance, suppressing excessive TNF-α production – highlighting a complex, context-dependent role in inflammation^50–51^. In chronic inflammatory diseases, however, the net effect of sphingolipid dysregulation is detrimental: ceramide accrual is strongly associated with endothelial dysfunction, oxidative stress, and progression of cardiometabolic disorders^52–55^. Reducing ceramide synthesis or enhancing its catabolism has accordingly been shown to alleviate inflammation and metabolic pathology^56–58^. These insights into ceramides underscore the broader principle of lipid–protein molecular cross-talk in chronic disease: metabolic lipids serve as signaling messengers that can profoundly influence protein kinase cascades, transcription factors, and cell–cell communication within inflamed tissues.

Given the multi-layered and dynamic nature of these disturbances, the application of omics techniques to the study of mechanisms and diseases involving inflammation-related feedback requires profiling multiple molecular types (e.g., metabolome, lipidome and proteome) across multiple time points to reveal the dynamic behavior of the interactions between different molecules. Strategies to impute such profiles mutually have been proved helpful^59^. However, even when such profiles (experimental or predicted) exist, it is necessary to derive strategies allowing to use them to make the temporal interactions between different molecular levels apparent.

With this goal in mind, we implemented a metabolomics driven integrative multi-omics strategy, specific for longitudinal data series, to capture the temporal dynamics of systems-level dysregulation in two disease models in which inflammatory feedback plays an important role. By concurrently profiling the metabolome, lipidome, and proteome at multiple time points, our approach tracks how metabolic and signaling networks become “decoupled” during chronic inflammation. Traditional single-omics studies often fall short in delineating this decoupling – for instance, changes in metabolite levels may not concord with enzymatic or protein expression changes due to post-translational regulation and feedback loops^60^. Here, we leveraged computational tools including OMINT (an omics integration network analysis tool)^59^, LIPEA (Lipid Pathway Enrichment Analysis platform)^61^, Enrichr (an interactive gene-set enrichment utility)^62^, and MetaboAnalyst 6.0 (a suite for metabolomic and multi-omic data interpretation)^63^ to extend the metabolomic layer (represented by the differentially expressed metabolites at different time points) to proteomic and lipidomic layers, and subsequently do functional enrichment of all layers. This integrative framework allows identification of cross-talk between lipid/metabolite and protein networks and detection of pathway-level alterations that are not evident from one data type alone. Notably, recent studies have demonstrated the power of combined proteomic–metabolomic analyses in toxicology and metabolic disease, revealing mechanisms that would be missed by either omics approach in isolation^64–65^. By applying such a holistic strategy, we aimed to uncover how inflammatory signals and metabolic pathways become uncoupled using two studies that provide longitudinal metabolic data for two models of exemplary but complementary diseases with a dynamic inflammatory response: chronic Duchenne muscular dystrophy (DMD) and acute Bothrops snake venom injury. DMD is a genetic muscle disease that manifests chronic sterile inflammation, oxidative stress, and profound metabolic alterations in skeletal muscle and beyond^66^. Such metabolic alterations are seen in reduced levels of glycine, arginine, and proline in affected tissues^67,68^. In contrast, Bothrops envenomation causes an acute, toxin-driven inflammatory injury marked by systemic metabolic disturbance and tissue damage^69^. Despite their differences, these two models, spanning the acute time scale (Bothrops envenomation, hours) and the chronic time scale (DMD, weeks), involve intense inflamma-metabolic stress, making them ideal for testing our multi-omics framework across chronic and acute contexts.

## Materials and methods

### Dataset construction

We compiled two previously published metabolomics time-course datasets to serve as the basis of this study. The first dataset originates from a Bothrops envenomation model described by Wase et al.^69^. In that study, groups of 4 mice were injected with Bothrops asper venom and blood samples were collected at 1, 3, 6, and 24 h post-injection (with non-envenomed controls). Differentially expressed metabolites were derived from untargeted metabolomics analyses of plasma. For this dataset, metabolite peak intensities were median normalized and autoscaled. The p-value was computed using a linear model with covariate adjustment on the time-series data, with the control group (non-envenomed mice) as the reference. Changes in metabolites were considered significant if they had an adjusted p-value ≤ 0.05. The paper notes that applying a p-value ≤ 0.01 identified 122 significantly changed metabolites for B. asper envenomation (3 biological replicates per time point). The second dataset is a longitudinal Duchenne muscular dystrophy (DMD) model using 4 mdx and 4 control mice, reported by Tsonaka et al.^66^. Plasma metabolomics was profiled at 6, 12, 18, 24, and 30 weeks of age in dystrophic mdx mice versus wild-type controls. This dataset captured progressive changes in metabolite levels reflecting disease progression in the mdx model. Differential expression of metabolites was calculated from p-values using a t-test applied to log-scaled intensity metabolite levels. The p-value estimates the probability that the observed change between wild-type (WT) and mdx conditions occurred by chance. The experiment included 4 replicates per condition at each time point (106 metabolites × 4 replicates, except at timepoints 24 and 30 weeks where WT had 2 replicates), which provides stable means and reduces the false discovery rate. Therefore, a p-value ≤ 0.05 was used as the threshold for statistical significance, indicating a ≤ 5% probability that the observed difference is due to random chance alone.

### Methodology of the strategy used in deriving functional terms in all other layers (protein and lipid) from differentially expressed metabolite data

**Fig. 1:**
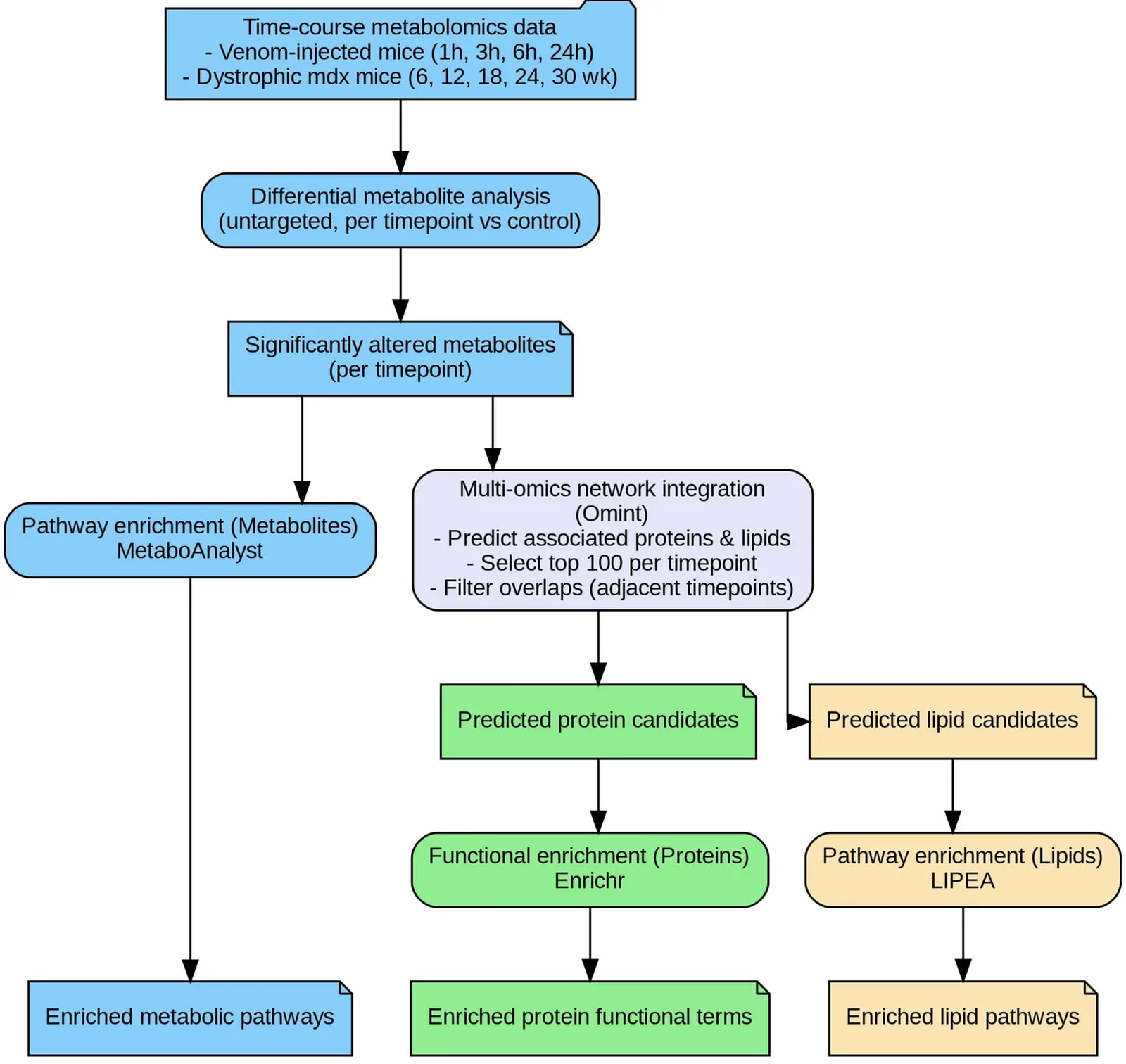
Workflow for metabolite-driven multi-omics integration. Time-course metabolomics datasets from the venom-injected mouse model and the dystrophic *mdx* mouse model are each processed independently to identify significantly altered metabolites at each timepoint. Metabolite-level pathway enrichment is performed using MetaboAnalyst. Differential metabolites are then projected onto the protein and lipid layers using OMINT, which ranks associated molecules by network proximity and selects the top 100 candidates per timepoint. These ranked molecules are aggregated into putative protein and lipid sets, after which molecules appearing in adjacent timepoints are removed to retain only dynamically changing species. The resulting protein candidates undergo functional enrichment with Enrichr, while lipid candidates are analyzed using LIPEA, yielding metabolite-, protein-, and lipid-level functional pathways for each model.

We employed a computational multi-omics integration strategy to infer functional insights across the proteomic and lipidomic layers using the metabolic changes as a starting point, as shown in Fig. 1. The overall approach was to leverage known biochemical relationships and enrichment analysis tools to “project” differential metabolite signals onto the protein and lipid domains. First, the list of significantly altered, differentially expressed (DE), metabolites at each time point was submitted to pathway enrichment analysis using MetaboAnalyst. This yielded a set of enriched metabolic pathways or metabolite-centric functional terms for each time point (e.g., aminoacyl-tRNA biosynthesis, arginine/proline metabolism), effectively summarizing the primary biological processes perturbed at the metabolite level.

**Fig. 2:**
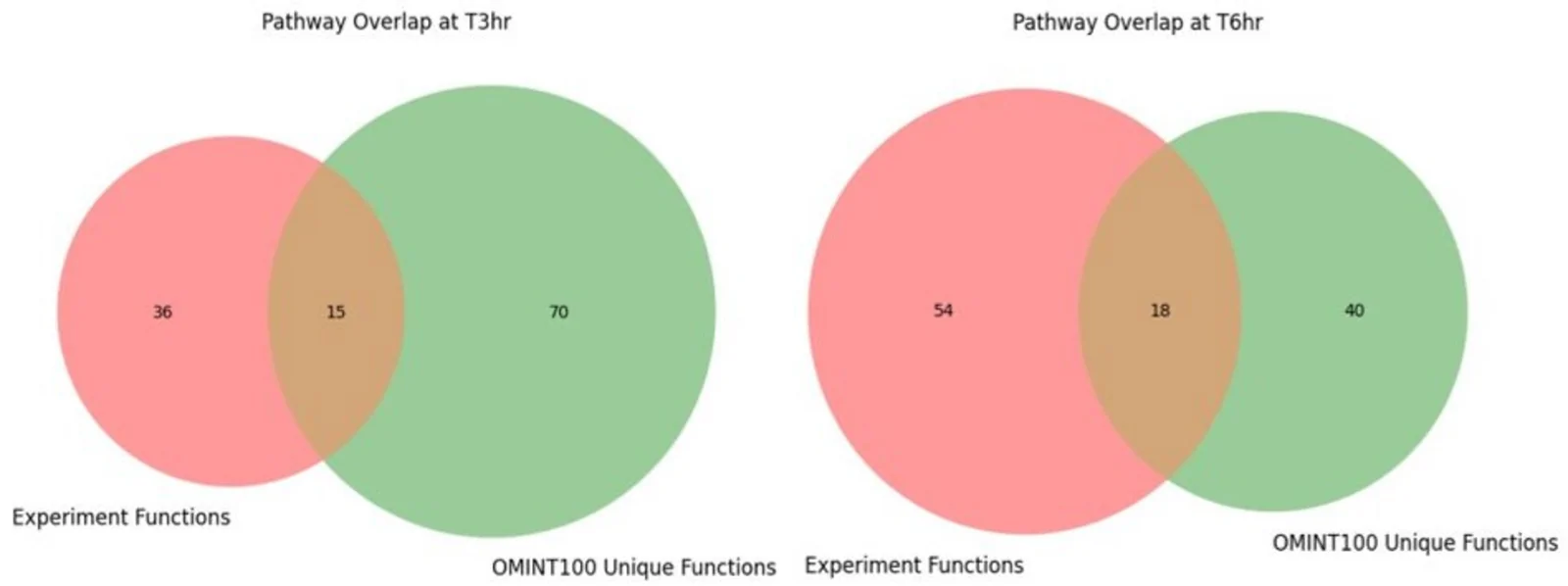
Validation of metabolomics-driven pathway predictions via Venn diagram overlap analysis in the Bothrops envenomation model. For each timepoint (3 h, 6 h post-venom), the set of enriched metabolite pathways inferred from the lipidomic data (OMINT-based projection) is compared to the set of pathways directly enriched from the metabolomic data. The Venn diagrams (one per timepoint) illustrate the common pathways (overlap) and unique pathways from each approach. All timepoints exhibit a significant intersection, indicating that the integrative method recapitulates many of the same biological processes identified by traditional metabolomics. This overall agreement supports the validity of the cross-omic integration strategy. The experimental part (Experiment Functions) for the functional enrichment of metabolites was obtained from Suppl data 8, while the predicted part (omint100 unique functions) was obtained from Suppl data 15, to generate this figure. A similar comparison could not be performed with the DMD model data because only metabolomic data was provided in the corresponding study.

**Fig. 3:**
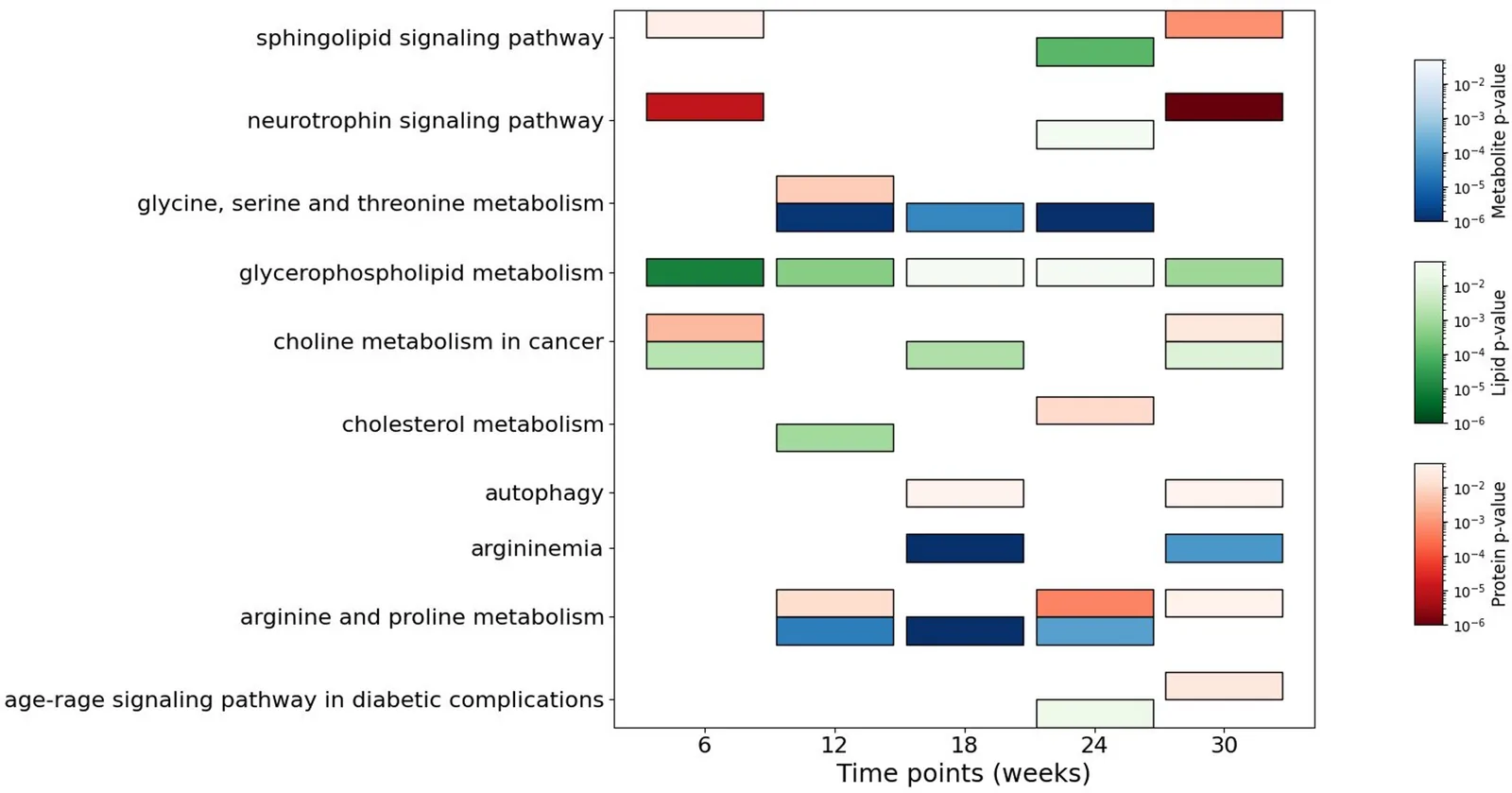
Time-resolved multi-omics analysis of the DMD (mdx) mouse model. Key pathway dynamics are illustrated across 5 time points (6, 12, 18, 24, and 30 weeks). Sphingolipid (SP) and neurotrophin (NT) signaling pathways show early protein-level activation at 6 weeks (e.g., NT effectors TRAF6, CALM1/3; SP effectors PPP2CA, NOS3, with TP53 common to both) and a delayed lipid-level response by 24 weeks (accumulation of ceramide and other sphingolipids). By 30 weeks, both pathways converge at the proteomic level (sharing KRAS, NOS3 and other nodes), indicating extensive cross-talk. Arginine and proline metabolism is depicted with early metabolite accumulation (elevated arginine, citrulline from 6 weeks; ornithine by 12–18 weeks) and a lagging protein response (enzymes like OAT and NOS3 upregulated by 30 weeks), corresponding to the development of argininemia and altered nitric oxide signaling. The AGE–RAGE signaling pathway is absent in early/mid stages but emerges at 30 weeks in the proteome (involvement of NOS3, FN1, KRAS), coinciding with prior ceramide buildup at 24 weeks. An autophagy-related response is shown activating at 18 weeks (appearance of autophagy-linked proteins such as VAPA/VAPB) followed by lipid metabolic disturbances at 24 weeks (in cholesterol esters and phosphatidylcholine species). Symbols and color-coding indicate the omics layer of each molecule (metabolites, lipids, proteins) and highlight the temporal progression of pathway activation in chronic muscular dystrophy. This figure was generated from Suppl data 4, 5, and 6.

**Fig. 4:**
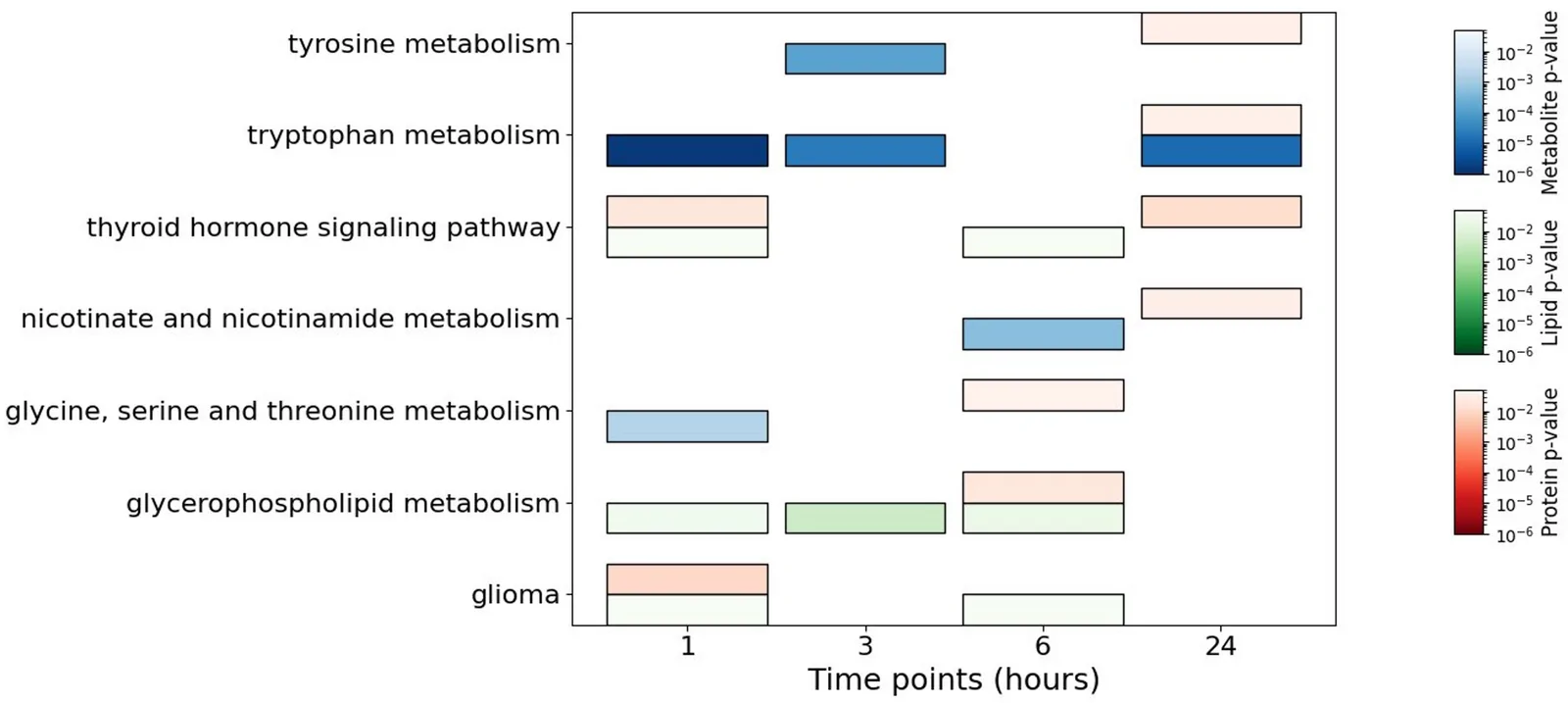
Time-resolved multi-omics analysis of the acute Bothrops envenomation model. Changes are mapped across 1, 3, 6, and 24 h post-venom. Tryptophan–kynurenine and nicotinamide (NAD⁺ salvage) pathways show immediate metabolite perturbations (within 1–3 h), whereas corresponding enzymes (e.g., DDC, AOX1, SIRT3) are induced only by 24 h, indicating a delay between metabolic stress and proteomic response. Thyroid hormone signaling exhibits a biphasic pattern: an early phase at 1 h coupling high tyrosine levels with activation of stress-related proteins (TP53 stabilized via MDM2 modulation, and CTNNB1), followed by a transient decoupling (no new proteins at 3–6 h despite sustained tyrosine) and a late phase at 24 h characterized by induction of transcriptional regulators (MYC, ESR1) independent of substrate. The glycine/serine/threonine metabolism pathway is rapidly engaged; by 1 h post-venom, glycine and serine levels drop (consumed for antioxidant defense), and by 6 h key enzymes (e.g., SHMT2, CHDH) are upregulated to support glutathione synthesis and stress mitigation. Glycerophospholipid metabolism is acutely impacted by venom PLA₂ activity: elevated lysophospholipids (LPA, LPC) are detected at 1–3 h, reflecting membrane phospholipid hydrolysis, while partial compensatory responses appear by 6 h (e.g., increased CHKB and ACHE indicating membrane repair and neuromuscular adaptation). By 24 h, both metabolite and protein changes begin to resolve, highlighting the transient nature of the acute response. This figure was generated from Suppl data 10, 11, and 12.

Next, we utilized the OMINT integrative network tool to extend the longitudinal DE metabolomics signatures to the other omic layers. OMINT is a recently developed multi-omics association methodology that can be used to link metabolites with corresponding lipids and proteins (see^59^ for details). Shortly, the associations are based on an interaction network of proteins, lipids and metabolites generated from known physical, enzymatic and genetic connections, constructed so that the geometrical positions of the nodes (proteins, lipids and metabolites) reflect similarities in their connectivity, ultimately connected to similar biological functions. This strategy is grounded in prior multi-omics integration methodologies that seek to connect molecular layers through shared biology. For example, Blum et al. (2022) introduced a network-based framework to deduce metabolic pathway changes from proteomic data alone, by leveraging known metabolite–protein interactions and expanding traditional pathway models^70^. Our OMINT approach follows a similar strategy of using established genetic and biochemical networks as a bridge between omics: in our case, starting from metabolomics and predicting cross-omic effects. In practice, for each set of DE metabolites at a given timepoint (computed using the p-value from individual samples in control and “diseased” conditions, as described in "Dataset construction") in our data, OMINT identifies a set of proteins (such as enzymes, transporters, or other gene products) and a set of lipids (for that timepoint) ranked by their proximity in the underlying network to a few of the metabolites in the list (closest three by default). The top 100 molecular species ranked by OMINT are then selected. This step effectively generates a “projected” list of molecules in the protein and lipid layers that are likely to be impacted given the observed metabolite changes. We aggregated all such protein candidates into a putative DE protein set, and similarly compiled all associated lipids into a putative DE lipid set for each time point. To focus on the molecules (proteins or lipids) with the most dynamic changes, we then removed all instances of molecules present in adjacent timepoints. This filtering strategy is motivated by established time-series omics analyses, which demonstrate that contrasts between adjacent time points are particularly informative for identifying regulatory drivers and state transitions, whereas persistently dysregulated molecules often reflect downstream or compensatory effects rather than active regulators^71–73^. We assume that molecules with stable values have low probability of driving the pathology. Accordingly, this filter prioritizes stage-specific molecular changes that are more likely to underlie temporal reprogramming of biological functions. We acknowledge that this approach may exclude molecules that remain consistently dysregulated throughout the disease course; however, such molecules are implicitly captured across multiple time points, whereas the present strategy emphasizes dynamic transitions between disease stages rather than sustained baseline abnormalities.

To derive functional annotations from the resulting predicted protein and lipid lists, we performed set enrichment analyses using appropriate tools. Genes (from protein candidate lists, extracted using UniProt) were analyzed for functional enrichment with Enrichr, a web-based platform that accepts gene lists and returns over-represented functional terms from various libraries. In parallel, the lipid candidate lists were analyzed with LIPEA (Lipid Pathway Enrichment Analysis), a specialized tool for over-representation analysis of lipid signatures. LIPEA mapped the list of altered lipids to KEGG lipid metabolism pathways and other lipid-related pathways. By this multi-step procedure, the initial DE metabolite data were translated into three sets of functional terms – one for each omics layer (metabolite, protein, and lipid) – all derived essentially from the metabolite changes but through a computational projection onto other layers. All enrichment analyses (MetaboAnalyst for metabolites, Enrichr for proteins, and LIPEA for lipids) were performed using default parameters and significance criteria as recommended by the tool developers. Enriched terms with adjusted p-value ≤ 0.05 were considered significant in our study. The outcome of this multi-omics enrichment pipeline was a collection of inferred functional terms in the proteomic and lipidomic layers, all driven by the metabolite-level data via the OMINT strategy.

### Benchmarking of the strategy: comparing to functional terms enriched from experimentally derived differentially expressed metabolites at individual time points

To evaluate the biological plausibility of our metabolite-driven functional projection, we performed a post-hoc functional validation by comparing OMINT-predicted metabolite functions—inferred from experimentally differentially expressed lipids—with functions obtained by direct enrichment of experimentally measured differentially expressed metabolites. Overlap between predicted and observed functional terms was visualized using Venn diagrams (Fig. 2; Suppl. Data 13, and 14).

This benchmarking focused on mid-stage time points (3 and 6 h), which capture stabilized metabolic reprogramming and provide the most interpretable window for cross-omic functional concordance. Early (1 h) and late (24 h) time points are dominated by transient signaling or secondary adaptation, respectively, which can obscure causal lipid–metabolite relationships. At the mid-stage time points, we observed substantial functional overlap, supporting the biological plausibility of OMINT predictions.

## Results

### Time-resolved multi-omic analysis of DMD evolution

To study the dynamics and possible cross-talk between the metabolite, lipid and protein layers in DMD, we obtained DE metabolites from a metabolomic profile time series at five time points (weeks 6, 12, 18, 24 and 30; Suppl. Data 1 from^66^ ) and applied OMINT to infer DE proteins and DE lipids (top 100 molecules each, Fig. 1). Because the proteomic and lipidomic layers are predicted from metabolite data, all protein and lipid changes described below represent inferred associations and should be interpreted as hypothesis-generating. To focus the set of predicted molecules on those with the most dynamic changes, the lists were filtered by removing occurrences found in adjacent time points (Supplementary data 2 and 3, respectively; see Methods for details). Set functional enrichment analysis (see Methods for details) was applied on the sets of DE molecules for each time point, experimental (metabolites; Suppl. data 4) or predicted (proteins and lipids; suppl. data 5 and 6, respectively) to get relevant biological functional terms associated with the molecular layers.

#### Inferred sphingolipid signaling and neurotrophin cross-talk in DMD

Our temporal multi-omics analysis suggests a coordinated, phased interplay between sphingolipid and neurotrophin signaling pathways during DMD progression (Fig. 3; see Suppl. data 4, 5 and 6 for full list of enriched terms and corresponding molecules), characterized by inferred early proteomic initiation, mid-phase lipid remodelling, and late-stage pathway convergence. At the experimentally observed metabolite layer, neurotrophin and sphingolipid signaling were not directly detected, highlighting the multi-omic inference. At the OMINT-predicted protein layer (Suppl. data 5), neurotrophin signaling was inferred to emerge at week 6 via MAP3K3, TRAF6, GRB2, CALM1/2/3, and TP53, and was again inferred at week 30 with CALM1/2/3 and KRAS. Sphingolipid signaling was inferred to appear concurrently at week 6 (PPP2CA, NOS3, TP53) and expanded by week 30 to include TRAF2 and KRAS alongside core components. In contrast, the predicted lipid layer showed delayed activation at week 24, with neurotrophin signaling inferred through ceramide (C00195), while sphingolipid metabolism, a precursor to signaling, featured dihydroceramide (C12126), ceramide (C00195), and sphingomyelin (C00550) (Suppl. data 6).

Based on these inferred associations, three distinct phases of pathway overlap appear to delineate the progression of molecular cross-talk over time. At week 6, the overlap is inferred primarily at the proteomic level, where both neurotrophin and sphingolipid signaling are predicted to share TP53 as a common regulatory node but diverge in their downstream effectors. Neurotrophin signaling is inferred to preferentially engage the TRAF6–MAP3K3–CALM1/2/3 axis, whereas sphingolipid signaling is inferred to be characterized by activation of the PPP2CA–NOS3 branch. By week 24, the overlap is predicted to shift to the lipid layer, with ceramide (C00195) emerging as a central molecule linking both pathways, accompanied by additional contributions from dihydroceramide and sphingomyelin within the sphingolipid metabolic network. At week 30, pathway cross-talk is inferred to become most pronounced at the proteomic level, with convergence through shared KRAS signaling and coordinated involvement of CALM1/2/3 in neurotrophin signaling alongside TP53, NOS3, and TRAF2 in sphingolipid signaling, suggesting extensive integration between the two pathways. We hypothesize that, mechanistically, early neurotrophin proteins (TRAF6, CALM1/2/3) reflect Trk/p75NTR receptor activation of Ras/MAPK and Ca²⁺/calmodulin cascades, initiating pro-survival and inflammatory responses^74^. We further hypothesize that p75NTR triggers ceramide generation via sphingomyelinases, explaining the week 24 lipid surge^75^. Ceramide and its derivative sphingosine-1-phosphate (S1P) form a rheostat balancing apoptosis and survival^76^. In dystrophic muscle, chronic infiltration of immune cells leads to sustained production of pro-inflammatory cytokines such as TNF-α and IL-1β, which are known to activate sphingomyelinases; we propose that activation of sphingomyelinases increases ceramide generation, thereby shifting the ceramide–S1P balance toward pro-apoptotic signaling. Thus, this inflammation-driven bias in sphingolipid signaling provides a hypothetical mechanistic link between persistent immune activation and progressive myonecrosis in DMD^77,78^. Also, the inferred late-stage enrichment of NOS3 (in sphingolipid signaling) aligns with dystrophic nitric oxide dysregulation, linking lipid signaling to vascular and metabolic dysfunction. Finally, the week 30 inferred proteomic convergence suggests ceramides (week 24) prime a cross-talk (from week 6) that amplifies KRAS-mediated signaling (week 30).

This inferred temporal architecture: early proteomic initiation to mid-phase lipid switch to late integrated signaling, suggests sphingolipid-neurotrophin cross-talk as a potential modulator of dystrophic progression. We hypothesize that the ceramide-S1P rheostat at week 24 represents a critical control point for inflammation and survival decisions. If validated, therapeutic targeting of ceramide metabolism (e.g., sphingomyelinase inhibitors), S1P signaling, or NOS3 activity could disrupt stage-specific pathology, offering precision strategies for DMD.

#### Protein-level dynamics suggests the appearance of an argininemia-like profile in DMD

Our temporal multi-omics analysis indicates a decoupling between metabolic and inferred proteomic adaptations in arginine and proline metabolism during DMD pathogenesis, characterized by early metabolite accumulation, which precedes the secondary functional emergence of an argininemia-like signature at the proteomic layer (in week 30) (Fig. 3; Suppl. data 5). At the observed metabolite layer, pathway activation emerged at week 6 with differential levels of arginine and citrulline and a dynamic addition of ornithine and proline through weeks 12, 18, and 24 (Suppl. data 4) when the predicted proteomic layer is engaged (week 12 and week 24). This early dysregulation is consistent with the hypothesis of impaired nitric oxide (NO) synthesis due to neuronal nitric oxide synthase (nNOS) mislocalization in dystrophic muscle, diverting arginine flux toward ornithine and proline production by arginase enzyme^79^. The inferred protein layer (Suppl. data 5) showed delayed engagement, first appearing at week 12 with GATM (guanidinoacetate N-methyltransferase), OAT (ornithine aminotransferase), and ALDH2 (aldehyde dehydrogenase 2). This 6-week lag suggests that the initial metabolic dysfunction occurs without detectable transcriptional/translational compensation, although protein-level changes may be below the OMINT prediction sensitivity. At week 30, the profile narrowed to OAT and endothelial nitric oxide synthase (NOS3), which we interpret as a potential signature of persistent ornithine metabolism and attempted NO pathway restoration^80^. Absence in the predicted lipid layer (Suppl. data 6) further supports amino acid-centric dysregulation.

We propose that the initial metabolite-protein disconnect reflects post-translational nNOS inhibition dominating early disease, with arginine catabolism shifting toward urea cycle intermediates and proline. As a hypothesis, late-stage elevated metabolites suppress nitric oxide synthase activity (evident in the emergence of NOS3 at week 30), which may contribute to a neurotoxicity profile reminiscent of argininemia (present at proteomic level in week 30)^81^. These inferred protein associations need independent proteomic validation.

#### Inferred AGE–RAGE signaling reflects dataset limitations at the metabolite layer in DMD

Advanced glycation end-product–receptor for AGE (AGE-RAGE) signaling pathway in diabetic complications was inferred exclusively at the protein layer during late-stage DMD progression (week 30), driven by NOS3, FN1, and KRAS (Fig. 3, Supp. data 5). This pathway is absent at early and mid-stages (weeks 6–24) and undetectable at the observed metabolite layer, which is expected because advanced glycation end products (AGEs) are reactive, form stable protein adducts, and are often poorly captured in metabolomics due to their size, charge, and inherent instability. As a result, AGE-RAGE activity may be more readily detected by inference at the proteomic rather than metabolite layer. The predicted lipid layer shows ceramide (C00195) accumulation at week 24, preceding the inferred AGE-RAGE proteomic activation and converging with adipocytokine signaling, necroptosis, neurotrophin signaling, and sphingolipid metabolism. This observation raises the hypothesis that ceramide acts as a pleiotropic lipid integrator of membrane remodeling, apoptosis, and inflammation^23,82^. Based on prior knowledge, high levels of ceramide can be a result of RAGE activation. Specifically, the AGE-RAGE pathway can trigger increased ceramide production, which may contribute to various health issues^83^. RAGE-ceramide axis has also been implicated as a necessary contributor to AGE-mediated disrupted cardiomyocyte mitochondrial function^84^.

#### Inferred associative response of cholesterol and choline metabolism via autophagy-mediated membrane remodeling

The regulation of autophagy involves both choline and cholesterol via distinct pathways, which are frequently annotated in pathway databases under cancer-related categories due to their extensive characterization in proliferative stress models, but represent conserved stress-response mechanisms also active in muscle degeneration. As a phosphatidylcholine (PC) precursor, choline participates in autophagosome biogenesis and the mTOR signaling cascade. Cholesterol, however, modulates autophagy dynamics by affecting membrane properties, particularly within the context of chaperone-mediated autophagy (CMA), and additionally impacts the mTOR pathway^85–87^. Our OMINT-inferred results show that autophagy at week 18 precedes cholesterol metabolism at week 24 in the proteomic layer. Cholesterol metabolism is predicted to implicate VAPA, VAPB and VDAC1 (Suppl. data 5), which are interconnected proteins that contribute to autophagy by influencing membrane contact sites, particularly between the ER and mitochondria. Their interactions are hypothesized to regulate critical steps in autophagosome formation and calcium signaling, which are essential for proper autophagic function^88^. Prior to week 24, cholesterol metabolism (C02530: cholesterol ester, C00422: Triacylglycerol, C00187: cholesterol) appeared in week 12 at the predicted lipid layer (Fig. 3). Autophagy plays a complex role in lipid homeostasis by both breaking down and potentially influencing the synthesis of triglycerides and cholesterol^85,89^. Taken together, these inferred results suggest how the interplay between proteomic and lipidomic layers of the cholesterol pathway may be affected by autophagy during dysregulation. The dysregulation of autophagy in the proteomics of mdx mouse has been reported by Armengol et al.^90^. Autophagy has also been presented as a therapeutic target in DMD^91^.

Furthermore, choline metabolism at the predicted lipid layer (C00157; Phosphatidylcholine, C04230: 1-Acyl-sn-glycero-3-phosphocholine) coincides with autophagy at the protein layer in week 18. Phosphocholine is a key intermediate in Phosphatidylcholine synthesis, and its levels can reflect the extent of membrane degradation during autophagy^92–93^. In week 30 both pathways converge at the predicted proteomic layer via KRAS (Suppl. data 5). We hypothesize that KRAS-mediated activation of the PI3K/AKT/mTOR pathway, extensively described in cancer biology, also represents a general stress-adaptive signaling mechanism that regulates autophagy and survival in non-proliferative tissues, including dystrophic muscle. We further speculate that, despite mTOR’s normal role as an autophagy inhibitor, this dysregulated signaling ultimately enhances autophagic activity, facilitating disease progression^94–95^.

### Time-resolved multi-omic analysis following Bothrops envenomation

To study the dynamics and possible cross-talk between the metabolite, lipid and protein layers in a model of Bothrops envenomation, we obtained DE metabolites from a time series at four time points (hours 1, 3, 6 and 24;^69^; Suppl. data 7) and applied OMINT to infer DE proteins and DE lipids (top 100 molecules each; Fig. 1). Again, all protein and lipid changes described are OMINT predictions and represent inferred associations. To focus the set of predicted molecules on those with the most dynamic changes, the list was filtered by removing occurrences found in adjacent time points (Supplementary data 8 and 9, respectively; see Methods for details). Set functional enrichment analysis (see Methods for details) was applied on the experimental sets of DE molecules for each time point, experimental (metabolites; Suppl. data 10) or predicted (proteins and lipids; Suppl. data 11 and 12).

#### Inferred coordinated dynamics of tryptophan and nicotinate/nicotinamide metabolism in NAD⁺-related metabolic pathways

Dysregulation of tryptophan and nicotinate/nicotinamide metabolism has been shown in DMD. Tryptophan is a vital amino acid that is primarily metabolized through the kynurenine pathway, which also plays a role in inflammation and immune responses. In DMD, this pathway is altered, with specific focus on kynurenine (KYN) and NAD⁺^96–98^. In the context of toxic envenomation, our time-resolved multi-omics analysis suggests that the proteomic response to Bothrops in NAD⁺-related pathways is both delayed and pathway-specific, in contrast to the rapid metabolite-level shifts observed in the early hours post-venom (Fig. 4). At the inferred protein layer, tryptophan metabolism shows no detectable change from 1 to 6 h after envenomation, despite strong observed metabolite perturbations at 1–3 h. Notably, it was inferred to emerge only at 24 h (DDC, AOX1; Suppl. Data 11), coinciding with a metabolite-level reappearance, suggesting a late-phase proteomic engagement of enzymes in this pathway. The interplay between these two enzymes (DDC, AOX1), along with other factors in NAD metabolism, could influence cellular responses to various stresses, including changes in oxygen availability^99–100^. We therefore hypothesize that this temporal lag reflects that initial NAD⁺ production via tryptophan catabolism is primarily metabolite-driven at earlier timepoints, with protein-level regulation being a downstream adaptation, possibly reflecting delayed synthesis or post-translational activation of key enzymes invisible to inferred expression proteomics. Likewise, nicotinate and nicotinamide metabolism were predicted to exhibit an exclusive proteomic signal at 24 h (AOX1, SIRT3; Suppl. Data 11), with no earlier protein-level inference. This follows its appearance at the metabolite level by 6 h, suggesting a two-step salvage pathway activation, initially driven by substrate availability (nicotinamide/niacin) and later supported by enzyme-level regulation. We propose that this late proteomic engagement may consolidate the NAD⁺ salvage pathway as the dominant mechanism once tryptophan catabolism wanes. These inferred dynamics support the hypothesis that both tryptophan and nicotinamide metabolic pathways converge at 24 h through AOX1 to fortify NAD⁺. Our data also suggest how proteomic changes may serve to stabilize and sustain pathway activity after the rapid, metabolite-driven adjustments of the acute phase. These findings underscore the need for integrating proteomics with metabolomics to fully resolve the temporal architecture of the envenomation response and the dual-route strategy for NAD⁺ biosynthesis, and remain to be validated with direct protein measurements.

#### Inferred coupling and decoupling of lipid–protein dynamics in the thyroid hormone signaling pathway

Our time-resolved multi-omics analysis of Bothrops envenomation suggests a distinct bi-phasic pattern of engagement in the thyroid hormone signaling pathway, characterized by an early lipid–protein coupling phase and a delayed, partially decoupled protein response (Fig. 4). At the inferred protein layer (Suppl. Data 11), pathway activity is first detected at 1 h, driven by the predicted regulatory genes MDM2, CTNNB1, and TP53, the latter being a transcriptional hub with known cross-talk between stress signaling and thyroid hormone receptor modulation^101–102^. The TP53 tumor suppressor is inhibited and destabilized by MDM2, however, under stress conditions, this downregulation is relieved and eases the accumulation of biologically active TP53^103^. This coincides with lipid-layer detection of thyroid hormone synthesis (Suppl. data 12), represented by L-tyrosine (C00165) predicted at 1 h. Notably, MDM2 is phosphorylated on tyrosine residues by the tyrosine kinase c-Abl^103^, leading us to hypothesize that the initial proteomic activation aligns with tyrosine mobilization at the lipid layer. By 3 h and 6 h, the observed metabolite layer (Suppl. data 7) shows sustained tyrosine availability alongside modified derivatives, γ-glutamyltyrosine and N-fructosyl tyrosine, and, by 6 h, oxidative modification to 3-nitro-L-tyrosine. At 6 h, the predicted lipid layer still contains L-tyrosine (C00165), maintaining precursor supply, yet no new proteins are recruited to the pathway at this timepoint, indicating a transient decoupling between lipid/metabolite availability and proteomic regulation.

A second proteomic activation phase of thyroid hormone signaling pathway was inferred at 24 h (with MYC, TP53, ESR1, and ACTB; Suppl. data 11) in the absence of detectable thyroid hormone synthesis lipids or tyrosine derivatives at the metabolite layer (Suppl data 12 and 7, respectively), and the simultaneous presence of tyrosine metabolism at 24 h at the predicted proteomic layer (Suppl. data 11). Thus, we hypothesize that tyrosine availability at 6 h in the lipid layer (preceded by tyrosine metabolism at 3 h at the metabolite layer, Suppl data 10) acts as a precursor for its metabolism at the proteomic level at 24 h. These inferred dynamics lead us to propose that in Bothrops envenomation, the thyroid hormone signaling pathway transitions from an early substrate-driven activation toward a late-stage transcriptional regulatory role, reflecting the broader metabolic shift from acute-phase hormone synthesis to longer-term cellular adaptation.

### Centrality of glycine, serine and threonine metabolic pathway in response to chronic and toxic inflammation

Our integrated analysis reveals distinct temporal dynamics in the glycine, serine, and threonine metabolism pathway across acute (Bothrops envenomation) and chronic (DMD) inflammatory conditions, with layer-specific patterns underscoring its potential mechanistic importance. In Bothrops envenomation, observed metabolite-level perturbations emerged within 1 h (Fig. 4, Suppl. data 10), preceding inferred protein-level engagement (Suppl data 11). Most proteomic changes were predicted to surface only at 6 h, driven by SHMT2 (serine hydroxymethyltransferase 2) and CHDH (choline dehydrogenase). This delayed protein response suggests rapid metabolite depletion for antioxidant defense^104^ preceding enzyme regulation. Oxidative stress has been shown to be induced by Bothrops venom^105^.

Conversely, in chronic DMD inflammation, sustained observed metabolite-level activation persisted from week 12 to week 24 (Fig. 3), while inferred protein-level shifts emerged exclusively at week 12 via GATM (glycine amidinotransferase), SHMT2, and CBS (cystathionine beta-synthase) (Suppl data 5). SHMT2 is an essential enzyme in the metabolism of one-carbon unit and catalyzes the conversion of serine and tetrahydrofolate (THF) to glycine^106^. CBS supports transsulfuration, linking serine to cysteine for glutathione synthesis to counter oxidative stress^107^. GATM modulates creatine biosynthesis^108^, possibly reflecting energy buffering demands in degenerating muscle. We hypothesize that the pathway’s acute-phase metabolite dominance (Bothrops) versus chronic protein-metabolite co-regulation (DMD) highlights its context-dependent role in inflammation resolution. Early glycine/serine depletion in envenomation may address immediate redox stress, while sustained engagement in DMD could integrate repair (one-carbon metabolism), antioxidant synthesis (transsulfuration), and energetics (creatine). These inferred temporal-layered activation patterns support the hypothesis of the pathway’s centrality as a metabolic hub adapting to inflammatory duration and severity. It is also pertinent to note how SHMT2 emerges as a common inferred hub for chronic and toxic inflammation.

### Centrality of Glycerophospholipid metabolic pathway in response to chronic and toxic inflammation

Our multi-omics analysis reveals divergent temporal dynamics in glycerophospholipid metabolism between acute (Bothrops envenomation) and chronic (DMD) inflammatory conditions, with predicted lipid-layer perturbations dominating both models. In Bothrops envenomation, glycerophospholipid metabolism was inferred to emerge immediately at the lipid layer (1 h: LPA [C04230], LPC [C04233], PA [C00641]), intensified at 3 h (adding G3P [C04438] and PA [C05973]), and persisted at 6 h (Fig. 4; Suppl data 12). This early lipid remodeling is consistent with venom phospholipase A₂ (PLA₂)-driven hydrolysis of membrane phospholipids^109^. Protein-level engagement was predicted to be delayed until 6 h, featuring ACHE (acetylcholinesterase), PHOSPHO1 (phosphoethanolamine/phosphocholine phosphatase), and CHKB (choline kinase beta) (Fig. 4; Suppl. data 11). These enzymes regulate choline metabolism and phospholipid synthesis, suggesting a hypothetical compensatory membrane repair following initial venom-induced degradation.

In chronic DMD inflammation, predicted lipid-layer disturbances persisted from week 6 to week 30 without protein-level enrichment at any timepoint (Fig. 3; Suppl. data 6). Early stages (weeks 6–12) featured LPA [C04230], LPC [C04233], and phosphocholine [C00157], indicative of ongoing membrane breakdown. By week 30, cardiolipin [C03819] and phosphatidylglycerol [C18126] emerged, which we interpret as a signal of mitochondrial membrane dysfunction^110–111^, a hallmark of dystrophic progression. The absence of inferred protein-level adaptations suggests defective enzymatic regulation of glycerophospholipid homeostasis, potentially exacerbating membrane instability in degenerating muscle.

Based on these inferred patterns, we propose that the pathway’s temporal architecture reveals fundamental differences in inflammatory resolution: Acute toxicity (Bothrops) exhibits rapid lipid hydrolysis (PLA₂-driven) followed by protein-mediated membrane repair, while chronic disease (DMD) shows progressive lipid-layer dysregulation without compensatory protein responses, leading to sustained membrane damage and mitochondrial dysfunction. If experimentally confirmed, this contrast would underscore glycerophospholipid metabolism as a critical determinant of inflammatory outcomes, where failed proteomic adaptation in chronic settings perpetuates tissue degeneration.

## Discussion

Integrating longitudinal metabolomic data with inferred proteomic and lipidomic changes provided a unified perspective of the molecular dynamics underlying both chronic and acute disease progression. Because all protein and lipid changes were computationally projected from metabolite measurements, the following interpretations are hypothesis-generating and require independent experimental validation. Using metabolites as the entry layer enabled the reconstruction of upstream protein perturbations and downstream lipid signaling, offering an inverse yet complementary view to traditional proteomics-led integration approaches. Central to this framework is the concept of multi-layer “decoupling,” which we define here as a temporally structured mismatch between molecular layers, whereby changes in metabolites or lipids precede, occur independently of, or diverge from corresponding protein-level regulation. Importantly, decoupling does not imply a lack of biological coordination, but rather reflects delays, buffering, or reprogramming across regulatory layers that unfold over disease time. This temporal and multi-layered approach suggests how different biological systems, chronic dystrophic muscle degeneration versus acute envenomation, may deploy shared molecular themes but at divergent speeds and with distinct adaptive outcomes.

In the chronic DMD model, the multi-omics patterns point toward a gradual but orchestrated remodeling of signaling and metabolic processes. Early engagement of neurotrophin and sphingolipid signaling suggests that dystrophic muscle rapidly activates compensatory pro-survival and inflammatory cascades, while the delayed accumulation of ceramide and related sphingolipids is consistent with a subsequent lipid-mediated modulation of stress signaling. This temporal separation between early proteomic activation and late lipid accumulation exemplifies a biologically meaningful decoupling event, in which lipid mediators accumulate independently of immediate protein-level control. This sequence: early protein activation, mid-phase lipid remodeling, and late convergence, leads us to hypothesize that muscle tissue initially attempts to preserve viability through signaling cross-talk but ultimately shifts toward chronic inflammation and apoptosis once lipid mediators accumulate. Functionally, this decoupling may bias the ceramide–S1P rheostat toward pro-apoptotic signaling, a shift that is further reinforced by chronic inflammatory cues and cytokine-induced sphingomyelinase activation. This observation aligns with preclinical evidence showing that excess ceramide contributes to muscle degeneration, inflammation, and fibrosis, and that pharmacologic blockade of ceramide synthesis or sphingomyelinase activity ameliorates pathology in mdx mouse models^112,113^. Thus, our inferred data support the hypothesis that ceramide metabolism represents a feasible therapeutic target in muscular dystrophy and related degenerative conditions.

Beyond sphingolipids, additional decoupling events in DMD emerge in cholesterol and choline metabolism, where lipid-layer alterations precede or diverge from proteomic engagement of autophagy-related regulators such as VAPA, VAPB, and VDAC1. This pattern suggests that membrane lipid remodeling can occur without synchronized activation of protein-level autophagy control. Such decoupling may have direct pathological implications, as altered cholesterol and phosphatidylcholine composition could disrupt ER–mitochondrial contact sites, calcium handling, and autophagic flux, all of which are central to dystrophic muscle degeneration. In this context, decoupling is proposed to reflect a failure to restore coordinated homeostasis, allowing maladaptive lipid states to persist and exacerbate tissue damage.

By contrast, the acute Bothrops envenomation model captures a fundamentally different role for decoupling, one that appears to support short-term flexibility and recovery rather than chronic failure. The early and transient activation of tryptophan and nicotinamide pathways suggests an immediate push toward NAD⁺ replenishment, followed by delayed enzyme engagement for sustained recovery. Here, decoupling manifests as an initial substrate-driven metabolic response that precedes proteomic stabilization, supporting the hypothesis that early NAD⁺ production is governed by metabolite availability rather than transcriptional or translational control. This two-phase NAD⁺ dynamic parallels findings in muscle and neurodegenerative disorders, where boosting NAD⁺ via nicotinamide riboside, niacin, or SIRT1 activators has shown benefits in preserving tissue function^114,115^.

A similar temporal structure is observed in thyroid hormone signaling, which exhibits inferred transient decoupling between sustained tyrosine availability at the metabolite/lipid level and delayed proteomic activation of regulatory nodes. The early phase may reflect rapid metabolic mobilization under acute stress (via c-Abl–MDM2–TP53 signaling), while the later phase potentially involves transcriptional reprogramming mediated by MYC and ESR1. Together, these patterns suggest that decoupling in acute injury contexts could enable rapid buffering of metabolic stress before longer-term regulatory programs are engaged. Such comparisons lead to the hypothesis that acute systems prioritize recovery and stabilization, whereas chronic conditions, such as DMD, fail to resolve decoupling, leading to progressive degeneration.

Furthermore, our study underscores that the temporal dimension is critical when assessing multi-omics data in disease. The timing of pathway activation may dictate whether a response is adaptive or maladaptive. For instance, early engagement of antioxidant and membrane repair pathways appears adaptive in acute venom injury, whereas delayed or insufficient engagement of similar pathways in chronic dystrophy is hypothesized to contribute to pathology. In the latter case, our analysis of arginine metabolism revealed disrupted flux between nitric oxide synthase and arginase pathways, which mirrors prior findings in dystrophic muscle where arginase activity promotes fibrosis and NO supplementation supports muscle regeneration^116,117^. Therapeutic modulation of this axis, using arginase inhibitors or NO donors, has shown promise in correcting muscle repair deficits in preclinical models. These findings were made possible by the multi-omics integration approach, which goes beyond traditional single-omics analysis that might focus on one layer at a time. By explicitly resolving temporal decoupling across layers, our approach provides mechanistic insight into why molecular changes fail to translate synchronously across biological scales. Notably, our cross-projection method (using OMINT) allowed us to generate testable hypotheses about unmeasured layers. Many of these hypotheses aligned with known molecular biology (strengthening confidence in the method), and some revealed novel connections (such as the suggestion of AGE-RAGE activity in DMD or the detailed timing of thyroid pathway responses in venom injury). These results encourage the development of computational approaches to supplement data across omics-layers, particularly when the dynamics of pathological mechanisms involve cross-talk across omics-layers. Moreover, the predicted involvement of NAD⁺ salvage and sirtuin signaling pathways highlights the translational potential of NAD⁺ restoration strategies (e.g., niacin or SIRT1 agonists) in modulating muscle resilience across both acute and chronic injury conditions, though these therapeutic hypotheses require direct testing in future studies.

Nevertheless, this study has several limitations. First, our use of OMINT for molecular projection is based entirely on in silico inference from interaction networks (like already established methodologies^70,118^), without experimental validation of predicted relationships. Second, although overlap between predicted and experimentally enriched functional terms reached approximately 50% at mid-stage timepoints (as shown in Fig. 2), this incomplete concordance indicates that many predictions may not generalize across biological contexts or may be sensitive to network topology. Third, the lack of direct perturbation experiments, such as ceramide inhibition or NAD⁺ boosting, limits our ability to assign causal roles to predicted drivers. Fourth, the metabolomics data were derived exclusively from plasma, which provides a systemic snapshot but cannot resolve tissue-specific metabolic events in dystrophic muscle or at the venom injection site. Consequently, the observed metabolite changes may reflect contributions from multiple organs and systemic stress responses rather than local pathology, and the inferred protein and lipid associations may not accurately capture compartment-specific regulation. This limitation is particularly relevant for the interpretation of acute versus chronic inflammatory dynamics, where local tissue microenvironments may differ substantially from the plasma metabolome. In addition, plasma metabolomics alone cannot robustly inform tissue-specific inflammatory processes or disease etiology, which constrains the interpretation of acute versus chronic inflammatory dynamics and risks the over-interpretation of systemic metabolic signatures. Future studies combining tissue-targeted perturbations with temporally resolved multi-omics measurements will be essential to validate whether resolving maladaptive decoupling can alter disease trajectories. Finally, the small sample sizes in the original metabolomics studies (e.g., *n* = 5 per time point for the DMD model, and *n* = 3 for Bothrops envenomation) reduce statistical power and may limit the detection of subtle, yet biologically meaningful, changes. These limited sample sizes also increase the risk that some predicted differences are driven by biological outliers or technical variability rather than true disease progression effects. Moreover, the parametric p-values on which our differential metabolite calls are based (t-test and linear model) rest on distributional assumptions that may not be fully met with such small samples, making them sensitive to deviations from normality. For illustration, while a non-parametric test like Mann–Whitney U test, produces a substantially similar set of significantly changing metabolites for the first three time points of the Bothrops envenomation model, it fails to provide significant metabolites for the last two time points, which have a reduced number of replicates (Supplementary data 16 and 17). Therefore, our findings should be viewed as exploratory and hypothesis-generating, and confirmation in larger, independent cohorts with multi-tissue sampling is essential.

## Supplementary Information

Below is the link to the electronic supplementary material.

Supplementary Material 1

Supplementary Material 2

Supplementary Material 3

Supplementary Material 4

Supplementary Material 5

Supplementary Material 6

Supplementary Material 7

Supplementary Material 8

Supplementary Material 9

Supplementary Material 10

Supplementary Material 11

Supplementary Material 12

Supplementary Material 13

Supplementary Material 14

Supplementary Material 15

Supplementary Material 16

Supplementary Material 17

## Data Availability

All the data used are public and have been cited in the Materials and Methods section.
